# Our Exchanges

**Published:** 1883-05

**Authors:** 


### OUR EXCHANGES.

Will our exchanges who now send to the New York office of this journal, or to the former Medical Editor, kindly change the address and direct to the editor, at No. 11 West Chippewa Street, Buffalo, N. Y. We are well aware that it is a task to overhaul the books for such a purpose, but if they only knew how earnestly we desire it, and how much we dislike to lose a single number, we are sure that all our contemporaries would cheerfully comply, as we should be happy to do under like circumstances. Remember that *all* communications to The Independent Practitioner should now be sent to Buffalo.

